# Evaluation of a Dengue NS1 Antigen Detection Assay Sensitivity and Specificity for the Diagnosis of Acute Dengue Virus Infection

**DOI:** 10.1371/journal.pntd.0003193

**Published:** 2014-10-02

**Authors:** Laura L. Hermann, Butsaya Thaisomboonsuk, Yongyuth Poolpanichupatam, Richard G. Jarman, Siripen Kalayanarooj, Ananda Nisalak, In-Kyu Yoon, Stefan Fernandez

**Affiliations:** 1 Department of Medicine, University of Toronto, Toronto, Ontario, Canada; 2 Department of Virology, Armed Forces Research Institute of Medical Sciences, Bangkok, Thailand; 3 Viral Diseases Branch, Walter Reed Army Institute of Research, Washington D.C., United States of America; 4 Queen Sirikit National Institute of Child Health, Bangkok, Thailand; University of California, Berkeley, United States of America

## Abstract

**Background:**

Currently, no dengue NS1 detection kit has regulatory approval for the diagnosis of acute dengue fever. Here we report the sensitivity and specificity of the InBios DEN Detect NS1 ELISA using a panel of well characterized human acute fever serum specimens.

**Methodology/Principal Findings:**

The InBios DENV Detect NS1 ELISA was tested using a panel composed of 334 serum specimens collected from acute febrile patients seeking care in a Bangkok hospital in 2010 and 2011. Of these patients, 314 were found to have acute dengue by either RT-PCR and/or anti-dengue IgM/IgG ELISA. Alongside the InBios NS1 ELISA kit, we compared the performance characteristics of the BioRad Platelia NS1 antigen kit. The InBios NS1 ELISA Ag kit had a higher overall sensitivity (86% vs 72.8%) but equal specificity (100%) compared to the BioRad Platelia kit. The serological status of the patient significantly influenced the outcome. In primary infections, the InBios NS1 kit demonstrated a higher sensitivity (98.8%) than in secondary infections (83.5%). We found significant variation in the sensitivity of the InBios NS1 ELISA kit depending on the serotype of the dengue virus and also found decreasing sensitivity the longer after the onset of illness, showing 100% sensitivity early during illness, but dropping below 50% by Day 7.

**Conclusion/Significance:**

The InBios NS1 ELISA kit demonstrated high accuracy when compared to the initial clinical diagnosis with greater than 85% agreement when patients were clinically diagnosed with dengue illness. Results presented here suggest the accurate detection of circulating dengue NS1 by the InBios DENV Detect NS1 ELISA can provide clinicians with a useful tool for diagnosis of early dengue infections.

## Author Summary

Infections by the mosquito-transmitted dengue virus continue to increase, threatening the health of people in over a hundred countries worldwide. The lack of effective mosquito control, licensed dengue vaccines or specific therapeutics to treat dengue infections presents challenges to reduce the burden of this disease. Rapid and accurate diagnosis of dengue infections is critical for the reduction of patients' morbidity and mortality. We present data that support the use of the InBios DENV Detect NS1 ELISA for the detection of dengue NS1 in patient serum. The InBios NS1 ELISA kit was tested against sera collected from acute fever patients seeking medical care in a Bangkok, Thailand hospital during 2010 and 2011. The data demonstrate the InBios DENV Detect NS1 ELISA accurately detects circulating dengue NS1 in the tested specimens, demonstrating high sensitivity and specificity. Nonetheless, the sensitivity of the NS1 ELISA kits was found to vary depending on the serological status of the patient (primary versus secondary dengue infection), time of specimen collection and dengue serotype. Further, the performance characteristics of the InBios DENV Detect NS1 ELISA were found to meet and exceed those of the commonly used Platelia Dengue NS1 Ag kit.

## Introduction

Dengue fever (DF) and dengue hemorrhagic fever (DHF) are mosquito-borne illnesses caused by infection with four related, but antigenically-distinct, dengue viruses (DENV1, DENV2, DENV3, DENV4). The virus is thought to be responsible for close to 400 million infections per year worldwide, of which approximately 100 million are clinically apparent [1]. Although many dengue vaccines are currently under development, none have been licensed. Similarly, there are no specific licensed therapeutics against DF or DHF. The outcome of patients with DF or DHF depends significantly on early diagnosis, leading to appropriate clinical management. Currently, there is no US FDA-approved diagnostic test that can accurately detect dengue NS1 during the acute febrile stage of infection. Traditional serological diagnosis measuring levels of anti-dengue IgM and IgG is hampered by cross-reactivity especially in dengue endemic areas where more than one flavivirus co-circulate [2]. Moreover, serological approaches are based on the detection of antibodies, which can take several days to develop. RT-PCR is sensitive for diagnosis early in infection, but is relatively expensive and requires specialized equipment and skills that may not be available in resource-poor settings where dengue virus is endemic. An NS1 antigen capture ELISA, first developed in 2000 for DENV [3], was based on the premise it would act as a surrogate marker for viremia. Circulating NS1 in the serum of acute DF and DHF patients is an attractive target for diagnosis as it is a viral glycoprotein released from infected cells as soluble polymers as early as day 1 post-infection. It can remain circulating for several days after defervescence [4] and is detectable in the serum of patients with primary or secondary dengue infections [3]. A number of commercial kits have been developed and have been subjected to field evaluation [5]–[16] including a number of second-generation rapid assays for point-of-care use for early diagnosis of dengue infection [17]–[21]. The sensitivity and specificity of these assays vary significantly depending on the dengue serotype and lineage [21].

In this study, we estimated the diagnostic accuracy of the InBios DENV Detect NS1 ELISA (InBios, USA) and compared it to the widely used Platelia Dengue NS1 Ag kit (Bio-Rad, France). We used human serum specimens collected in Thailand during public health service testing during 2010 and 2011. These specimens were collected during acute febrile episodes and sent to our laboratories at the Armed Forces Research Institute of Medical Sciences (AFRIMS) for dengue laboratory confirmation. All specimens were tested using AFRIMS laboratory reference assays for dengue infection by IgM antibody capture ELISA and RT-PCR.

## Methods

### Clinical Specimens

Clinical specimens used in this study were collected through routine public health service testing in Bangkok, Thailand during 2010 and 2011 from 334 patients suspected to have dengue fever within 7 days from onset of symptoms (Table 1). Clinical diagnoses by local Thai clinical staff were based on the 2011 SEARO World Health Organization [22] definition of acute dengue infection. Serum samples from patients were transferred to AFRIMS, Bangkok, Thailand and were maintained at −70°C until tested. All laboratory investigations were carried out at AFRIMS by experienced technicians following standard operating procedures. The technicians performing and interpreting the assays were blind to other test results and to any clinical information on the patients. Approval for the use of these specimens was obtained from the Institutional Review Boards of the Queen Sirikit National Institute of Child Health and of the Walter Reed Army Institute of Research. No private or confidential information was collected.

**Table 1:** Summary of study population.

	Confirmed Dengue n = 314	Other Febrile Illness n = 20
**Variable**		
Median Age, years, (IQR^a^) Range	9 (6 – 12) 1 month – 24.7 yrs	7 (4 – 9) 7 months – 15 yrs
Male∶Female	144 ∶ 170	8∶12
Median Days from Onset of Illness, (IQR^a^)	4 (3 – 5)	4 (3 – 5)
**Confirmation of Dengue Diagnosis**		
RT-PCR only	3 (1.0%)	
Serology only	15 (4.8%)	
RT-PCR and Serology	296 (94.3%)	
**DENV Serotypes**		
DENV-1	96 (30.6%)	
DENV-2	95 (30.3%)	
DENV-3	96 (30.6%)	
DENV-4	12 (3.8%)	
Indeterminate^b^	15 (4.8%)	
**Serological Status**		
Primary	51 (16.2%)	
Secondary	260 (82.8%)	
Indeterminate^c^	3 (1.0%)	
**Clinical severity**		
Dengue Fever^d^	159 (50.6%)	
Dengue Hemorrhagic Fever^e^	141 (44.9%)	
Other^f^	14 (4.5%)	

a IQR – interquartile range.

b Represents subjects confirmed as dengue positive by serological testing only. The infecting serotype was unable to be determined since they were negative by RT-PCR.

c Represents subjects confirmed as dengue positive by RT-PCR only. Serological studies were not positive and primary or secondary infection could not be determined.

d Dengue Fever clinical diagnosis based on SEARO WHO guidelines [22].

e Includes patients clinically diagnosed with DHF with plasma leakage, DSS and deaths based on SEARO WHO guidelines [22].

f No diagnosis was given in 8 cases. Other clinical diagnoses included one each of bronchitis, gastritis, viral gastroenteritis, viral-induced thrombocytopenia, query rickettsial infection and nonspecific viral infection.

### Dengue Reference Testing

Paired acute and convalescent serum specimens were tested for anti-dengue IgM (MAC) and IgG ELISA, Japanese encephalitis virus [23], [24] and chikungunya [25] assays. Acute samples were also tested by nested RT-PCR for the presence of dengue virus and serotype identity [26], [27]. Although the ELISA assessment utilizes both acute and convalescent samples for infection status, only acute samples were used in this study for testing the InBios and Platelia Bio-Rad assays.

A patient was determined to have an acute DENV infection by identification of dengue virus genome by RT-PCR from an acute serum sample and/or detection of anti-DENV IgM antibodies by MAC and/or a ≥2 fold rise to ≥100 U in paired acute and convalescent samples [17], [28]. DENV IgM-positive cases were considered to be primary infections if the ratio of DENV IgM to IgG was ≥1.8 [23]. If the ratio was <1.8, it was considered a secondary infection. A sample with any positive criterion listed was used in the analyses as part of the composite positive control group except when discussing serotype where only RT-PCR positive results were used.

### NS1 Antigen Detection Assays

The InBios DENV Detect NS1 ELISA test is an assay for the detection of dengue virus NS1 antigen in human sera. Kits were provided by InBios (Seattle, WA). The test is based on the capture of NS1 antigen using a sandwich-type immunoassay and was performed by strictly following the instructions provided by the manufacturer. Briefly, 50 µL serum samples and controls were diluted 1∶2 with sample diluent buffer containing the secondary antibody and incubated at 37°C for 1 hr in microtiter plates pre-coated with anti-NS1 antibody. After washing, the wells were treated with a conjugate solution containing horse radish peroxidase (HRP) polyclonal antibody and incubated for 30 minutes at 37°C. Wells were washed and incubated with 3,3′,5,5′-tetramethylbenzidine (TMB) substrate solution in the dark at room temperature for 20 minutes. After addition of the stop solution, the optical density was read at 450 nm. The immune status ratio (ISR) was calculated from the ratio of the optical density of the test sample divided by the mean optical density of the cut-off control. ISR values ≥1 were considered positive for the presence of NS1 antigen.

In parallel, the same amount of each sample (50 µL) was assayed on the same day using the Platelia dengue NS1 Ag kit (Bio-Rad) according to the manufacturer's instructions. Briefly, 50 µL of sample and controls were diluted 1∶2 with sample diluent and combined with 100 µL of diluted HRP-labeled anti-NS1 monoclonal antibody. This solution was added to microtiter plates coated with anti-NS1 monoclonal antibodies and incubated at 37°C for 90 minutes. After washing, complexes between the capture antibody, NS1 and HRP-labeled antibodies were detected by a colorimetric reaction after incubation with TMB for 30 minutes. After the addition of a stop solution, the optical density of samples was read at 450/620 nm. A sample ratio was calculated by dividing the optical density of the test sample by the mean optical density of the cut-off controls. Sample ratios of <0.5, 0.5 to <1.0 and ≥1.0 were considered negative, equivocal and positive for the presence of NS1 antigen, respectively.

For data analyses, equivocal values were considered negative. Both the InBios and BioRad assays are not marketed as quantitative assays.

### Statistical Analysis

Test characteristics with their respective binomial 95% confidence intervals (CI) were calculated using standard formulas. Differences in assay performance were calculated by using McNemar's test [29]. Significance differences (p<0.05) in positivity rates relative to dengue virus serotypes were calculated using Pearson's chi-square or Fisher's exact test. SPSS for Windows version 19 and MedCalc version 12.4 software were used for analyses.

## Results

A total of 334 samples from individual subjects were evaluated (Table 1) from routine public health service samples collected in Bangkok, Thailand, between 2010 and 2011. The median age of the subjects was 9 years (range: 1 month to 24.7 years). DENV infection was confirmed in 314 (94.0%) subjects and serotype was determined by nested RT-PCR in 299 (95.2%). Fifteen (4.8%) subjects were negative by RT-PCR and confirmed as dengue positive by serological testing only. The serotype was unable to be determined in these 15 cases. Fifty one subjects (16.2%) had a primary infection, 260 (82.8%) had a secondary infection and serological status was not determined in 3 (1.0%) subjects as they were RT-PCR positive only. Clinical diagnoses by local Thai physicians were based on WHO SEARO 2011 guidelines [22]. There were 159 (50.6%) cases of DF and 141 (44.9%) cases of DHF/DSS with evidence of plasma leakage and/or deaths. Fourteen (4.5%) cases with laboratory confirmation of DENV infection were not given clinical diagnoses of dengue. No clinical diagnosis was given in 8 of the 14 subjects and there was one diagnosis each of acute bronchitis, acute gastritis, viral gastroenteritis, viral induced thrombocytopenia, viral illness and rickettsial illness. Sera samples were collected a median of 4 days from onset of illness (DOI). Twenty samples were negative for dengue infection.

Test performance characteristics of the InBios and Bio-Rad assays were compared against a composite reference standard including samples positive by RT-PCR positive and/or serological testing (Table 2). The overall sensitivity of the InBios assay was 86.0% (95% CI 81.7–89.4) and was significantly higher than the sensitivity of the Bio-Rad assay at 72.8% (95% CI 67.1–77.0) (McNemar's, p<0.0001). Both assays had specificities of 100% (95% CI 83.9–100.0) and there were no false positive results for either assay when compared to the composite reference standard. The InBios test was significantly more sensitive for patients ≤5 years of age at 95.1% compared to 83.8% for those >5 years (Chi-square, p = 0.02).

**Table 2:** Overall performance characteristics of the InBios and Bio-Rad assays compared to reference standard^a^.

	Sensitivity, % (95% CI^b^)	Specificity, % (95% CI)	Diagnostic Accuracy, % (95% CI)	PPV^c^, %	NPV^d^, % (95% CI)
**InBios**	86.0 (270/314)^e^ (81.7–89.4)	100.0 (20/20)^f^ (83.0–100.0)	86.8 (290/334)^g^ (82.8–90.0)	100 (270/270)^h^	31.3 (20/64)^i^ (21.2–43.4)
**Bio-Rad**	72.8 (227/314)^e^ (67.1–77.0)	100.0 (20/20)^f^ (83.9–100.0)	74.0 (247/334)^g^ (69.0–78.4)	100 (227/227)^h^	18.7 (20/107)^i^ (12.4–27.1)

a The composite reference standard included samples that were positive either by serology and/or RT-PCR.

b CI – confidence interval.

c PPV – positive predictive value.

d NPV – negative predictive value.

e Sensitivity = (true positives)/(total positive by reference standard).

f Specificity = (true negatives)/(total negative by reference standard).

g Diagnostic Accuracy = (true positives+true negatives)/(total number of samples).

h PPV = (true positives)/(total positive by InBios or Bio-Rad assay).

i NPV = (true negatives)/(total negative by InBios or Bio-Rad assay).

In Thailand, where the majority of presentations are secondary infections, we wanted to see if there was a difference in sensitivity between primary and secondary infections. The sensitivity of the InBios assay was 98.2% for primary infections (n = 51) and 83.5% for secondary infections (n = 260) which was significantly different (Chi-square, p = 0.002) (Table 3). This was not associated with a difference in the median day of presentation after illness onset which was 4 days for subjects presenting with either primary or secondary infection. The variation in sensitivity between primary and secondary infections was also seen with the Bio-Rad assay (96.3% vs 67.3% for primary and secondary infections, respectively; Chi-square, p<0.0001). There was no difference between the rate of NS1 detection for the InBios and Bio-Rad assays for primary infections, but there was a significant difference for secondary infections (McNemar's, p<0.0001). Sensitivity of NS1 detection with the InBios assay in IgG negative samples was 89.1% (251/282; 95% CI 85.0–92.2) and 55.2% (16/29; 95% CI 37.6–71.6) in IgG positive samples (Fisher's Exact Test, p<0.0001). A similar difference was also seen with the Bio-Rad assay with sensitivity of 77.2 (217/282; 95% CI 72.0–81.7) and 24.1% (7/29; 95% CI 12.2–42.1) for IgG negative and IgG positive samples, respectively (Fisher's Exact Test, p<0.0001). There was no difference in sensitivity for IgM positive or negative samples for either the InBios or Bio-Rad assays.

**Table 3:** Sensitivity of InBios and Bio-Rad assays differentiated by primary or secondary infection.

	Number of Specimens^a^ n	Median DOI^b^ (IQR^c^)	InBios Sensitivity (95% CI^d^)	Bio-Rad Sensitivity (95% CI^d^)
**Primary**	51	4 (3 – 6)	98.8% (89.7–100.0)	96.1% (86.8–99.0)
**Secondary**	260	4 (3 – 5)	83.5% (78.5–87.5)	67.3% (61.4–72.7)

a Total number of DENV positive samples is 314.

b DOI – days after onset of illness.

c IQR – interquartile range.

d CI – confidence interval.

**Figure 1:**
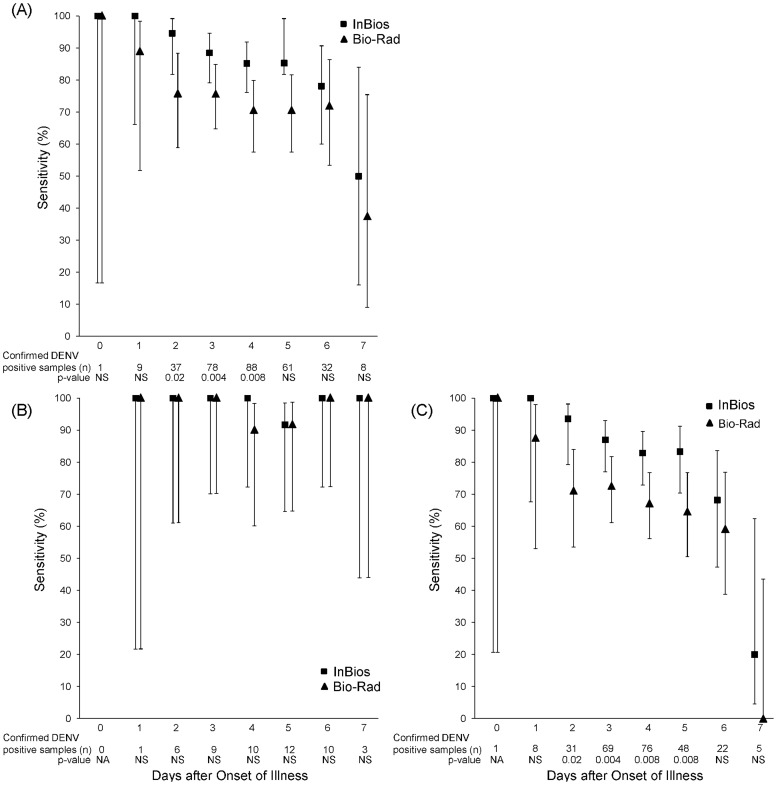
Sensitivity of InBios and Bio-Rad Assays stratified by date after onset of illness in (A) All infections and in (B) Primary and (C) Secondary infections. Serum samples (total samples, n = 314; primary, n = 51; secondary, n = 260) were tested using the InBios and Bio-Rad NS1 kits. Sensitivity was plotted against day post-onset of illness. p-values were calculated using McNemar's Chi-square test. NA – not applicable. Unable to do statistical analysis when value equals 0. NS – not significant.

When all positive samples (n = 314) were stratified by their DOI, the sensitivity of the test decreased at later time points (Figure 1A) for both the InBios and Bio-Rad assays dropping to 50.0% and 37.5%, respectively, by Day 7. The difference seen between the assays is primarily due to differences in the sensitivity for secondary infections (Figure 1C). The specificity of both assays was 100.0% throughout for both primary and secondary infections.

When considering primary infections, the rate of detection of NS1 antigen remained close to 100% for both the InBios and Bio-Rad assays for all days tested (Figure 1B). However, for secondary infections the InBios assay positivity remained above 90% through Day 2 after symptom onset and dropped to 68% by Day 6 (Figure 1C). In contrast, the sensitivity of the Bio-Rad assay decreased starting on the day following symptom onset. The InBios test was significantly more sensitive than the Bio-Rad assay from Day 2 through Day 5.

Performance characteristics also varied by serotype (Table 4). When evaluating PCR positive results only, differences in sensitivity were seen between serotypes (Fisher's Exact Test, InBios, p = 0.04 and Bio-Rad, p<0.0001). Detection of DENV-4 was least sensitive with the InBios assay, whereas detection of DENV-2 was least sensitive with the Bio-Rad assay. The InBios assay was more sensitive than the Bio-Rad assay for each serotype, but this difference was only significant for DENV-2 (McNemar's, p<0.0001).

**Table 4:** Sensitivity of InBios and Bio-Rad assays based on serotype.

Test	DENV-1 Sensitivity n = 96 (95% CI)	DENV-2 Sensitivity n = 95 (95% CI)	DENV-3 Sensitivity n = 96 (95% CI)	DENV-4 Sensitivity n = 12 (95% CI)
**InBios**	92.7% (85.7–96.4)	89.5% (81.2–94.2)	81.3% (72.3–87.8)	75.0% (46.8–91.1)
**Bio-Rad**	90.6% (83.1–95.0)	52.6% (42.7–62.4)	79.2% (70.0–86.1)	58.3% (32.0–80.7)
**p value**	0.48	<0.0001	0.6171	0.4795

Out of 334 samples, 301 subjects were given a clinical diagnosis of dengue infection. Only one of these patients was given an incorrect diagnosis of DF and 14 of the 314 subjects positive for dengue infection were not identified clinically. A small decrease in sensitivity was seen in primary infections between DF and DHF/DSS disease when using the BioRad assay (Table 5), but was not significant (Chi-square, p = 0.246).

**Table 5:** Sensitivity of InBios and Bio-Rad assays based on clinical diagnosis and serological diagnosis.

	Overall		Primary Infection		Secondary Infection	
Test	Dengue Fever^a^ Sensitivity n = 159	Dengue Hemorrhagic Fever^b^ Sensitivity n = 141	Dengue Fever Sensitivity n = 24^c^	Dengue Hemorrhagic Fever Sensitivity n = 22^d^	Dengue Fever Sensitivity n = 134^c^	Dengue Hemorrhagic Fever Sensitivity n = 118^d^
**DOI (mean)**	3.9	3.8	4.4	4.1	3.8	3.8
**InBios**	85.5% (79.2–90.2)	85.8% (79.1–90.6)	100.0% (86.2–100.0)	95.5% (78.2–99.2)	82.8% (75.6–88.3)	83.4% (76.2–89.4)
**Bio-Rad**	76.1% (68.9–82.1)	66.7% 58.5–73.9)	100.0% (86.2–100.0)	90.9% (72.2–97.5)	71.6% (63.5–78.6)	61.7% (52.9–70.1)

a Dengue Fever clinical diagnosis based on SEARO WHO guidelines [22].

b Includes patients clinically diagnosed with DHF with plasma leakage, DSS and deaths based on SEARO WHO guidelines [22].

c Total number of Dengue Fever equals 159, but 1 case could not be classified as primary or secondary infection.

d Total number of Dengue Hemorrhagic Fever equals 141, but 1 case could not be classified as primary or secondary infection.

## Discussion

No single diagnostic assay can accurately detect dengue infection throughout its clinical course. A number of commercial tests for diagnosis of dengue infection are available, but only two have received regulatory approval [30], [31], none of which measures NS1. Diagnosis of dengue is done by detection of genomic material by RT-PCR early during infection and with serological assays (detection of IgM and IgG) at later time points [22], [32]. In the last decade, NS1 antigen testing has become common for early diagnosis of dengue infection [5]–[21], [33]–[35]. However, negative NS1 test results late in infection does not rule out an acute dengue infection, as it may be caused by low circulating NS1 antigens in the blood [17], [36]. In this study we evaluated the InBios DENV Detect NS1 ELISA, which we compared against the widely used Bio-Rad Platelia Dengue NS1 Ag kit, for the diagnosis of acute dengue infection using hospital-based passive surveillance samples collected in Bangkok in 2010 and 2011. We compared it against the Bio-Rad assay since it was the first commercial NS1 antigen detection assay, is one of the best characterized systems and is also the most widely used NS1 assay in Bangkok, Thailand. We evaluated the overall sensitivity of the assays against a panel of acute specimens with known serological status (i.e. primary vs secondary infection), number of days after onset of illness, DENV serotype, and clinical diagnosis or severity of infection.

The performance of NS1 assays, alone and in combination with detection of IgM or IgG, have been extensively tested [8], [13], [17], [21], [37], [38]. While results are not always consistent amongst different cohorts and assays, a number of general comments can be made. Sensitivity is highest in primary infections, when testing occurs shortly after onset of symptoms and when IgG is not detectable. Variations in sensitivity are dependent on the comparator used and dengue serotype. In previous studies of NS1 antigen detection, the sensitivity varied between 34% and 96% [5]–[12], [14]–[21], [33]–[35] while the specificity was always very high. In our study, we found the overall sensitivities for the InBios and Bio-Rad assays were in keeping with previous studies evaluating the performance of the Bio-Rad assay [7]–[12], [15], [16], [19]–[21], [33]–[35]. A number of samples collected on DOI 6 were found to be NS1 antigen positive. They were RT-PCR negative yet were determined to be positive for dengue infection by serological testing. This discrepant result between the NS1 and RT-PCR assays is likely due to NS1 antigen circulating in the serum for longer periods than viral RNA; thus extending the diagnostic window beyond that for RT-PCR testing.

The sensitivity in primary infections was significantly higher for both the InBios and Bio-Rad assays as has been seen in a number of previous studies [5], [11], [12], [16]–[21], [33], [38] and cannot be explained by when samples were collected in relation to symptom onset. More primary infections were seen in subjects ≤5 years (40.9% vs 10.3% in subjects >5 years) and likely explains the increased sensitivity in this age group. As expected, we saw the overall sensitivity of both assays drop off for samples collected later after symptom onset [7]–[9], [11], [12], [14]–[17], [19]–[21], [33], [34], [38]. For the InBios assay, overall sensitivity remained above 80% until DOI 6. When results were separated into primary and secondary infections, the sensitivity for both the InBios and Bio-Rad assays remained above 90% throughout for primary infections. Thus, decreased overall sensitivity for samples tested at later time points was mostly due to the influence of the decreased sensitivity in secondary infections. Differences in sensitivity may also be explained by antibodies that bind to NS1which may be different in each kit, although this information is not available to us.

Detection of NS1 antigen in secondary infections may be hampered by a rapid rise in antibody levels due to the anamnestic antibody response [39] resulting in the formation of immune complexes likely with IgG antibody, which prevents the binding of capture or detection antibodies to NS1 antigen. Increased sensitivity was seen in studies where a step to dissociate immune complexes was included [7], [40]. In keeping with previous studies [7]–[9], [14], [33], [35], we found the sensitivity of NS1 detection in acute samples with the InBios assay and Bio-Rad assays was lower in IgG positive compared to IgG negative samples with no difference between IgM positive or negative samples. This parallels what we see in primary infections where there are low levels of IgG and higher sensitivities than in secondary infections with higher levels of IgG and lower sensitivities.

Differences in sensitivity between DENV serotypes have been seen in some [10], [12], [17]–[19], [21], [33]–[35], but not other studies [8], [9], [14], [20]. In our study, we saw differences in sensitivity between serotypes, but the profile was different between the two assays. As seen previously, the Bio-Rad assay was least sensitive to DENV-2 [10], [12], [20], [33]. This may be partially explained by a larger proportion of secondary infections (88.2%) in the panel of DENV-2 samples tested. However, this is less likely as the larger proportion of secondary infections in DENV-2 did not affect the sensitivity of the InBios assay which remained high (89.5%). For the InBios assay, sensitivity was highest for DENV-1 as seen previously for other NS1 kits [8], [9], [12], [18], [19], [21], [33]. Similar to other studies, sensitivity was lowest for DENV-4 samples in the InBios assay, although there were only a small number of samples for this evaluation [18], [21], [35]. A number of other groups have found the sensitivity to DENV-3 to be decreased [8], [17], [19], but this was not seen in the current study.

Results have not been consistent when evaluating the sensitivity of NS1 antigen detection based on disease classification. Some groups have found no differences [7], [34] whereas Osorio *et al.* [21] found a decrease in sensitivity to NS1 detection in more severe cases, but is thought to be because more severe cases tended to be secondary infections and presented at later time points than non-severe infections. In our study, no difference in sensitivity was seen for either the InBios or Bio-Rad assays based on clinical disease classification.

There were a number of limitations to our study. Samples for testing were chosen from archived samples from Thai patients and may not represent circulating dengue viruses elsewhere. Due to sample availability, only a limited number of primary infections and DENV-4 infections were included. Dengue negative samples were tested for Japanese Encephalitis and Chikungunya infections, but were otherwise not fully characterized. More comprehensive testing of the InBios assay and other NS1 antigen detection assays needs to be done and should incorporate a larger number of primary infections and samples collected from both children and adults. To date, no cross-reactivity has been seen with other flaviviruses [8], [41], but this finding needs to be confirmed in a larger cohort of acute phase samples with better characterized flavivirus-positive samples. Future studies also need to consider differences in geographic area, circulating serotype (and genotype), patient ethnicity, viremia, immunological response and clinical severity.

This report show the InBios DENV Detect NS1 ELISA has comparable, if not better, performance characteristics to other NS1 antigen kits. Although its sensitivity varies depending on the serological status of the patient, date of specimen collection and serotype of the infecting virus, its use for accurate diagnosis of dengue infection should be considered by clinicians especially early in infection.

## Supporting Information

Figure S1 **STARD checklist.**
(PDF)

Flowchart S1 **STARD flowchart for InBios DENV Detect NS1 ELISA.**
(PDF)

Flowchart S2 **STARD flowchart for BioRad Platelia.**
(PDF)
