# Supplementary material for: Evaluation of a Dengue NS1 Antigen Detection Assay Sensitivity and Specificity for the Diagnosis of Acute Dengue Virus Infection
Source: PLoS Negl Trop Dis. 2014 Oct 2;8(10):e3193. doi: 10.1371/journal.pntd.0003193 (PMC4183466; doi:10.1371/journal.pntd.0003193)
Supplement: Flowchart S1 — STARD flowchart for InBios DENV Detect NS1 ELISA. (PDF) [file pntd.0003193.s002.pdf]

STARD Flowchart for NS1 Platelia ELISA

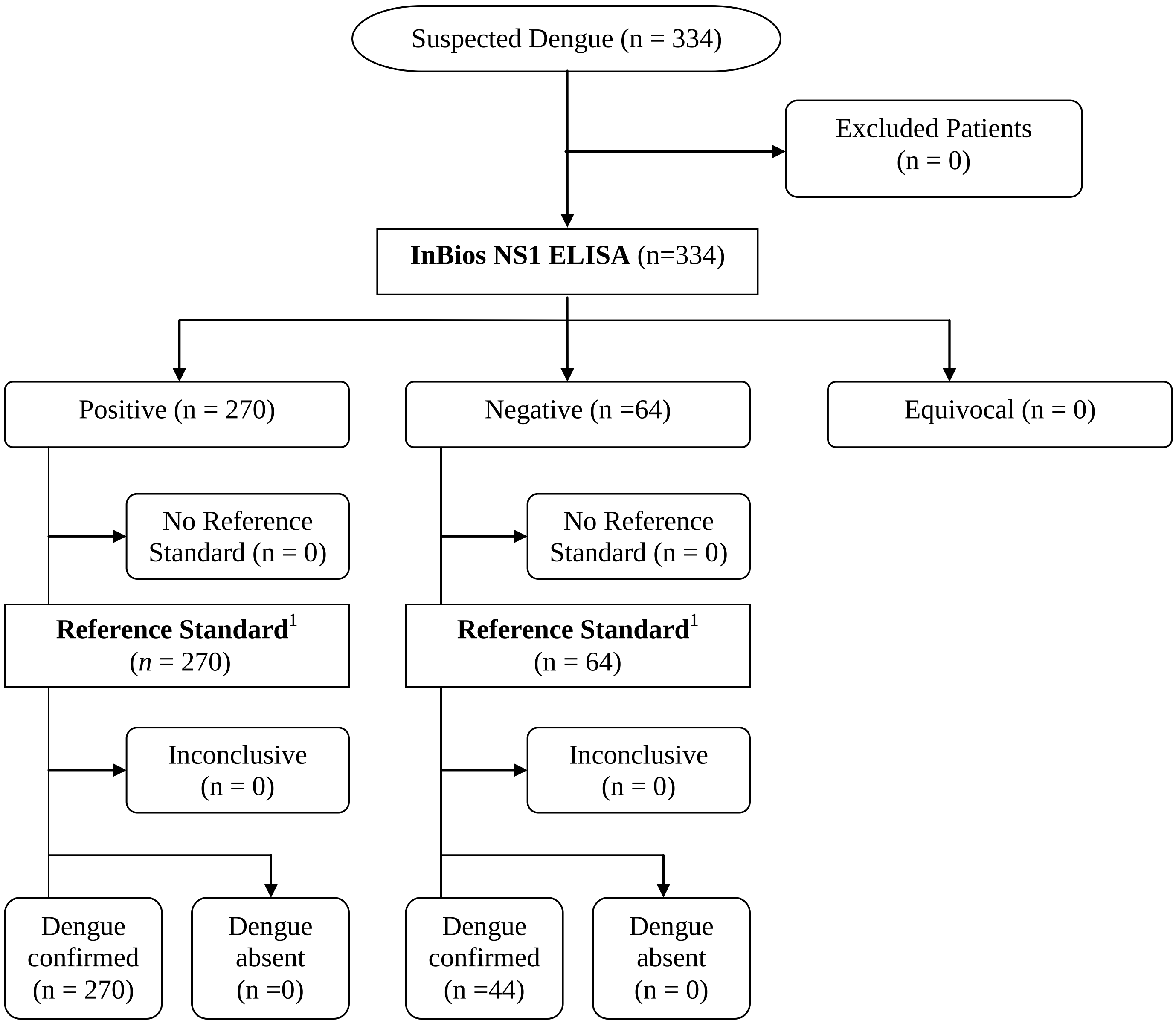

[1] A composite reference standard was used based on serological and RT-PCR testing.
