# Supplementary material for: Evaluation of a Dengue NS1 Antigen Detection Assay Sensitivity and Specificity for the Diagnosis of Acute Dengue Virus Infection
Source: PLoS Negl Trop Dis. 2014 Oct 2;8(10):e3193. doi: 10.1371/journal.pntd.0003193 (PMC4183466; doi:10.1371/journal.pntd.0003193)
Supplement: Flowchart S2 — STARD flowchart for BioRad Platelia. (PDF) [file pntd.0003193.s003.pdf]

STARD Flowchart for NS1 Platelia ELISA

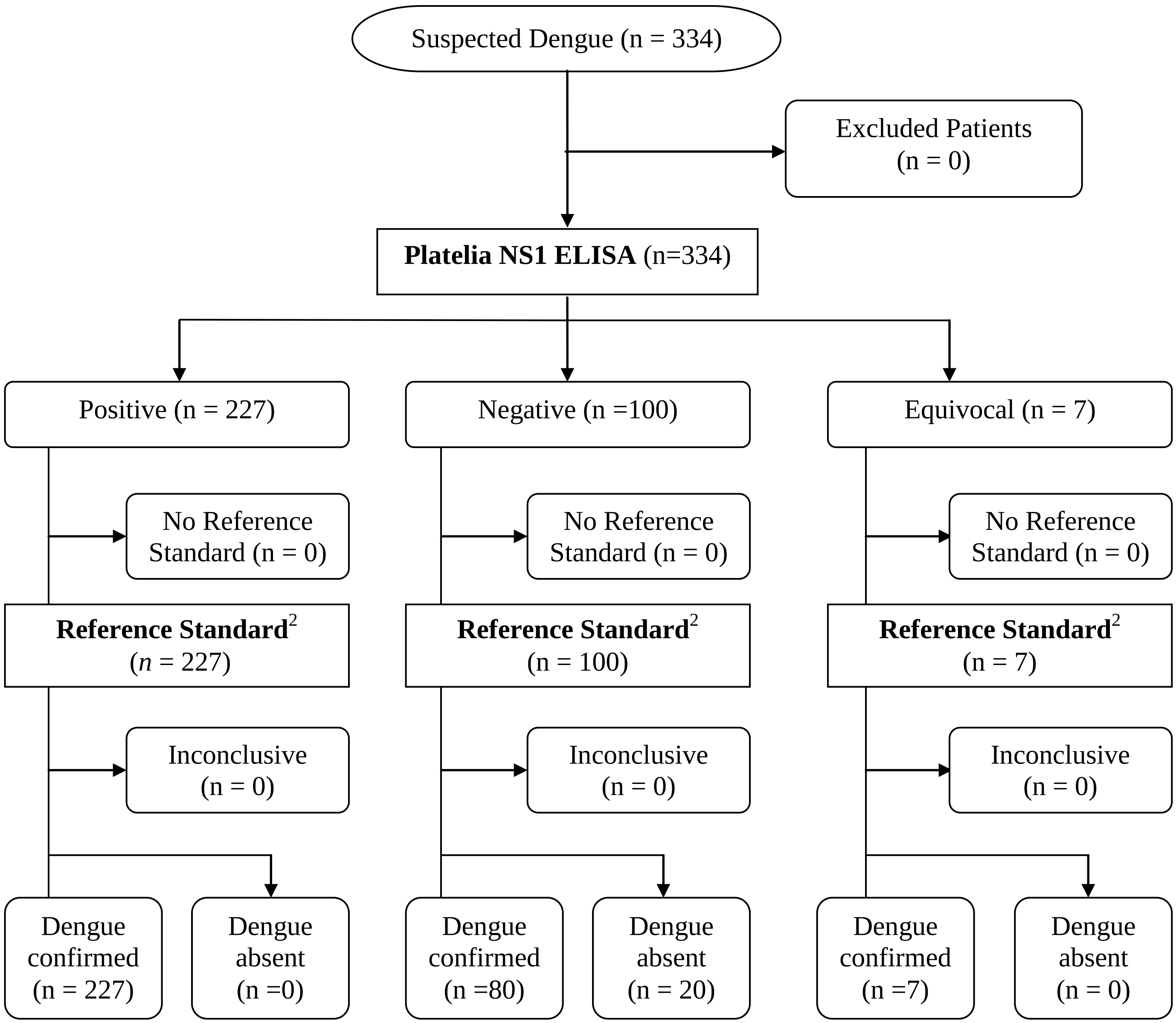

[1] An equivocal result was considered negative for data analyses.
[2] A composite reference standard was used based on serological and RT-PCR testing.
